# Non-invasive skin measurement methods and diagnostics for vitiligo: a systematic review

**DOI:** 10.3389/fmed.2023.1200963

**Published:** 2023-07-27

**Authors:** Parsa Abdi, Michelle R. Anthony, Christopher Farkouh, Airiss R. Chan, Amritpal Kooner, Simal Qureshi, Howard Maibach

**Affiliations:** ^1^Memorial University of Newfoundland, Faculty of Medicine, St. Johns, NL, Canada; ^2^College of Medicine, University of Arizona, Tucson, AZ, United States; ^3^Rush Medical College, Faculty of Medicine, Chicago, IL, United States; ^4^Division of Dermatology, Faculty of Medicine, University of Alberta, Edmonton, AB, Canada; ^5^Chicago College of Osteopathic Medicine, Midwestern University, Downers Grove, IL, United States; ^6^Division of Dermatology, Faculty of Medicine, University of California, San Francisco, San Francisco, CA, United States

**Keywords:** vitiligo, non-invasive techniques, diagnosis, imaging techniques, dermoscopy, reflectance confocal microscopy, ultraviolet light photography, wood's lamp

## Abstract

Vitiligo is a multifaceted autoimmune depigmenting disorder affecting around 0.5 to 2.0% of individuals globally. Standardizing diagnosis and therapy tracking can be arduous, as numerous clinical evaluation methods are subject to interobserver variability and may not be validated. Therefore, there is a need for diagnostic tools that are objective, dependable, and preferably non-invasive.

**Aims:**

This systematic review provides a comprehensive overview of the non-invasive objective skin measurement methods that are currently used to evaluate the diagnosis, severity, and progression of vitiligo, as well as the advantages and limitations of each technique.

**Methods:**

The Preferred Reporting Items for Systematic Reviews and Meta-Analyses (PRISMA) checklist was used for the systematic review. Scopus, Embase, Cochrane Library, and Web of Science databases were comprehensively searched for non-invasive imaging and biophysical skin measuring methods to diagnose, evaluate the severity of, or monitor the effects of vitiligo treatment. The risk of bias in included articles was assessed using the QUADAS-2 quality assessment scale.

**Results:**

An extensive literature search resulted in 64 studies for analysis, describing eight imaging techniques (reflectance confocal microscopy, computer-aided imaging analysis, optical coherence tomography, infrared photography, third-harmonic generation microscopy, multiphoton microscopy, ultraviolet light photography, and visible light/digital photograph), and three biophysical approaches (dermoscopy, colorimetry, spectrometry) used in diagnosing and assessing vitiligo. Pertinent information about functionality, mechanisms of action, sensitivity, and specificity was obtained for all studies, and insights into the strengths and limitations of each diagnostic technique were addressed. Methodological study quality was adequate; however, statistical analysis was not achievable because of the variety of methods evaluated and the non-standardized reporting of diagnostic accuracy results.

**Conclusions:**

The results of this systematic review can enhance clinical practice and research by providing a comprehensive overview of the spectrum of non-invasive imaging and biophysical techniques in vitiligo assessment. Studies with larger sample sizes and sound methodology are required to develop verified methods for use in future practice and research.

**Systematic review registration:**

(PROSPERO) database, (CRD42023395996).

## 1. Introduction

Vitiligo is a common autoimmune depigmenting disorder associated with the loss of functional melanocytes and melanin in the epidermis, typically presenting as circumscribed depigmented macules to patches (1). Currently, around 0.5 to 2.0% of individuals are affected with vitiligo worldwide, but appropriate medical care and research are limited compared to other dermatological pathologies (1, 2). Vitiligo does not exhibit a gender predilection, but it does appear to prefer specific anatomical locations, including the face and extensor surfaces (1, 3). The disease tends to manifest around 10 to 30 years, with a mean age of diagnosis around 15.6 years, and 70–80% of vitiligo diagnoses occur before age 30 (1). Although, vitiligo is typically asymptomatic and benign, the psychological and cosmetic consequences to patients may be overwhelming.

The current gold standard for diagnosing vitiligo relies on clinical examination, yet certain presentations, particularly early or evolving lesions, may be missed, necessitating additional diagnostic tools for confirmation. Various numerical scales also exist to assess outcome measurements for vitiligo, including the including the well-validated Vitiligo Extent Score (VES), the Self-Assessment Vitiligo Extent Score (SA-VES), and Vitiligo Area Scoring Index (VASI) (4–6). Commonly used diagnostic techniques for vitiligo include dermoscopy, Wood's lamp, digital photography with computerized image analysis, and invasive methods such as skin biopsies, however, several emerging, non-invasive techniques are currently being investigated.

Non-invasive methods offer distinct advantages over invasive approaches as they enable the longitudinal tracking of the same skin area without causing inflammation, irritation, damage, or other adverse reactions that could hinder accurate assessment. Despite the widespread use of several non-invasive and objective diagnostic techniques in vitiligo assessment, a comprehensive review of these methods is lacking. Therefore, the purpose of this systematic review is to provide a comprehensive overview of these non-invasive techniques, their strengths, limitations, and potential implications in the diagnosis and assessment of vitiligo. In the context of non-invasive techniques, our systematic review focuses on eight imaging techniques (reflectance confocal microscopy, computer-aided imaging analysis, optical coherence tomography, infrared photography, third-harmonic generation microscopy, multiphoton microscopy, ultraviolet light photography, and visible light/digital photograph), and three biophysical approaches (dermoscopy, colorimetry, spectrometry) used in diagnosing and assessing vitiligo.

## 2. Materials and methods

### 2.1. Study design

A systematic review was carried out using the Preferred Reporting Items for Systematic Reviews and Meta-Analyses (PRISMA) checklist and is registered in the International Prospective Register of Systematic Reviews (PROSPERO) database (CRD42023395996) (7).

### 2.2. Search strategy

Four electronic databases—Scopus, Embase, Cochrane Library, and Web of Science—were used for a comprehensive literature search. The search was based on studies that used objective, non-invasive imaging and biophysical skin measuring methods to diagnose, evaluate the severity of, or monitor the effects of vitiligo treatment. A literature search and examination of PubMed MeSH terms were used to obtain skin measurement diagnostic techniques. The following criteria included the principal search words: “vitiligo, diagnosis, and assessment.” An exhaustive list of all search terms used can be found in Supplementary material 1. If more data from the study was required, the authors were contacted. Additionally, a manual search was performed through the related articles' references list for relevant sources.

### 2.3. Eligibility criteria

Inclusion criteria included: (1) randomized controlled trials, non-randomized controlled trials, cohort studies, case series, and case reports; (2) studies that involved the assessment of cutaneous vitiligo (including segmental, nonsegmental, universal, generalized, mucosal, follicular, guttate, and hypopigmented vitiligo); (3) non-invasive objective imaging/biophysical tools for vitiligo diagnostic and measurement; (4) the investigation offered reliable data that could be studied, including the total number of participants and the insightful outcomes of each metric.

Exclusion criteria included: (1) invasive objective imaging/biophysical tools for vitiligo diagnostic and measurement; (2) research that did not offer adequate information on results in experimental or control groups; (3) *in vitro* and animal studies; (4) research reported in languages other than English; (5) meta-analysis, systematic reviews, and other reviews (not including primary source); (6) studies lacking full-text or only presenting abstracts.

**Figure 1:**
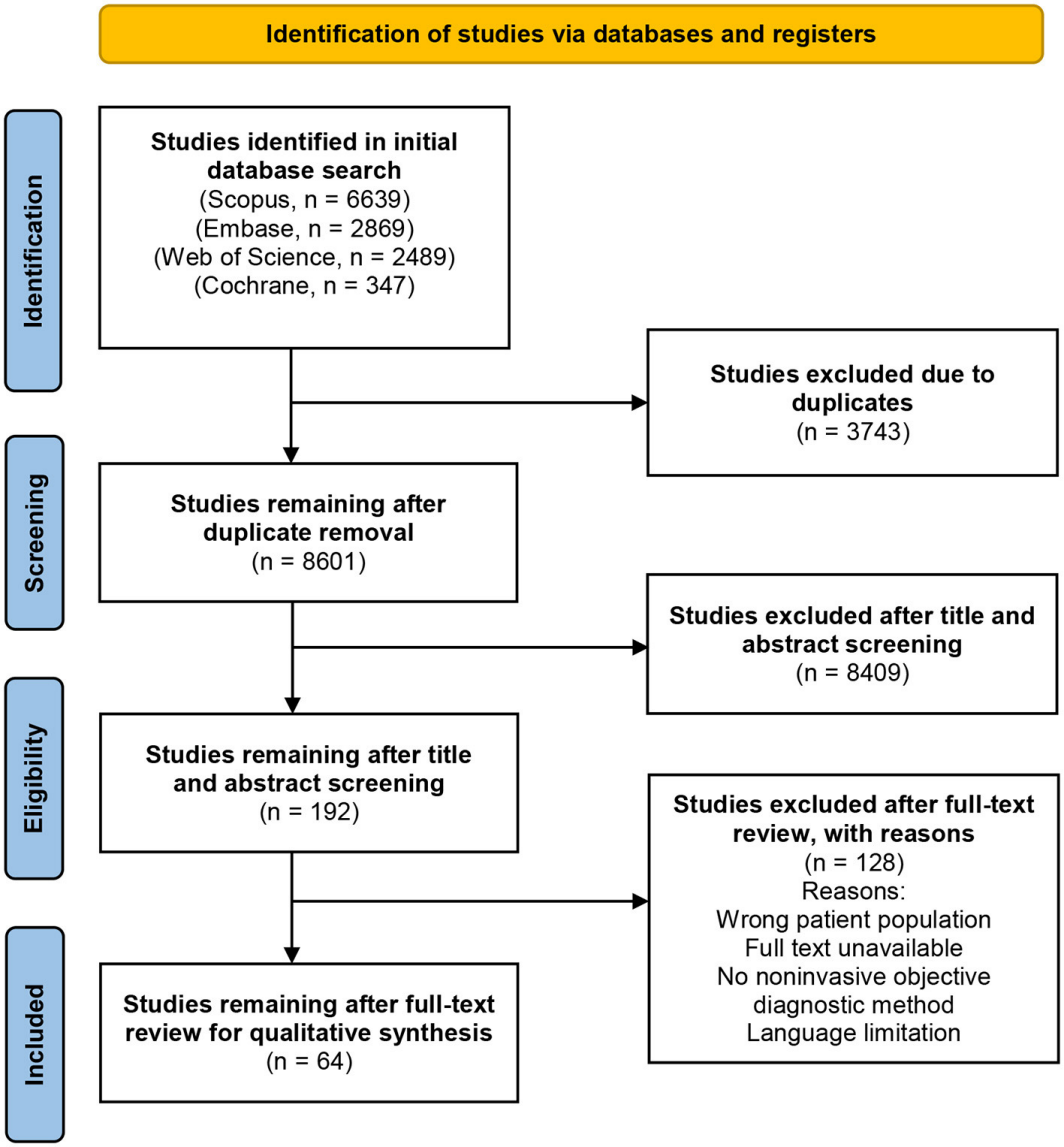
Process flow diagram of literature screening using the PRISMA guidelines.

The term “non-invasive” was defined as any procedure that theoretically cannot cause skin irritation, bleeding, or scarring. This criterion excluded biopsies, the epilation of eyelashes or hairs, the application of tape or glue to the skin, and the collection of excretions from sebaceous follicles or scrapings. Figure 1 depicts the PRISMA selection process flowchart used for this systematic review.

### 2.4. Data extraction

All four databases were searched to include published studies from inception until July 2, 2023. 8601 identified articles were independently reviewed for eligibility using the Covidence systematic review software by two authors (P. A. and M.A.) after duplicate removal from the list of publications. After an initial title and abstract screening, 192 articles underwent a full-text examination to determine their eligibility. A total of 64 studies remained after a full-text review for qualitative synthesis. Conflicts were settled through discussion or consulting a third, unbiased investigator (C.F). The study design, participant count, vitiligo classification, measurement sites, evaluated skin parameters, and study findings were extracted. Imaging techniques, and biophysical approaches were each given a narrative synthesis. Information about each method's advantages, limitations, and measurement principles was tabulated. Statistical analysis was not feasible due to the wide variety of tests evaluated and the non-standardized presentation of diagnostic accuracy outcomes.

### 2.5. Quality assessment

In this systematic review, the authors employed the Quality Assessment of Diagnostic Accuracy Studies (QUADAS-2) tool to assess the quality of the studies included (8). Two independent reviewers (P. A. and M.A.) evaluated the risk of bias, and any discrepancies were worked out through discussion or by consulting a third, unbiased investigator (C.F). The QUADAS-2 tool has four domains: patient selection, index test, reference standard, and flow and timing. Each domain has questions that assess the risk of bias and applicability of the study and addresses issues related to patient selection, index tests, reference standard validity, and potential bias in patient flow. The QUADAS-2 tool does not incorporate a quality score.

## 3. Results

### 3.1. Study characteristics

In total, 64 articles describing eight imaging modalities and three biophysical approaches utilized in diagnosing and evaluating vitiligo were included in this systematic review for analysis. Due to the employment of multiple diagnostics methods, several studies were included in numerous categories to ensure a holistic overview. Table 1 lists all imaging methods and biophysical skin measuring modalities considered for this review, along with the advantages and limitations of each technique.

**Table 1:** Summary table of imaging, biophysical and manual non-invasive techniques used in vitiligo diagnostics and therapy monitoring.

**Classification of method**	**Technique**	**Study design(s)**	**Measurement principle**	**Advantages**	**Limitations**
Imaging	Reflectance confocal microscopy (9–19)	10 cross-sectional; 1 case series	High-resolution cellular imaging of the epidermis and superficial dermis, based on the confocal principle	High-resolution; real-time imaging; surface imaging; multiplexing; fast procedure (10 minutes); portable system; cost-effective compared to another microscopy	Limited depth sensitivity; Lower signal-to-nose-ratio; requires smooth sample preparation; extensive training requirements for diagnostic interpretation; no z-axis imaging; sample specificity
	Computer-aided imaging analysis (20–37)	16 cross-sectional; 2 methodological; 1 prospective open label; 1 multiple-reader multiple-case diagnostic	Image processing using various methods including, ANN, CNN, DNN, PCA, ICA, fuzzy clustering, K-mean, GLCM, linear clustering	Comprehensive quantification; portable; mapping of facial distributions; machine learning capabilities; non-skin contact	Lack of imaging in z-axis; lacking real-time imaging; very expensive; extensive training requirements for diagnostic interpretation; novel techniques, without standard method
	Optical coherence tomography (38–41)	4 cross-sectional	Low-coherence interferometry to detect reflections of an infrared light source, which are used to create an image of the scanned tissue until the reticular dermis	Real-time imaging; rapid (2–10 min); cross-sectional imaging comparable to histology; deep surface penetration depth (1.5 mm); wide field of view; high spatial resolution (3–10 μm^2^)	Extensive training requirements for diagnostic interpretation; expensive; lower axial resolution than RCM; lower resolution at reticular dermis; lowered image quality in uneven skin; only architectural changes; limited subcellular details
	Infrared photography (42)	1 cross-sectional	Utilizes the infrared domain of the electromagnetic spectrum for tissue assessment.	Painless; easy to use; rapid; cost-effective; real-time results; objective diagnostics	Equipment sensitivity; limited information on deep structures; operator dependence; no differentiation between arterial and venous structures; training needed for image interpretation
	Multiphoton microscopy (43, 44)	2 cross-sectional	Pulsed lasers to excite multiple low-energy photons, allowing high-resolution visualization of melanocyte density and distribution	High-resolution; label-free; real-time imaging; deep tissue penetration; multiparametric imaging; preservation of tissue integrity	Restricted field of view; lack of standardization; small sample size
	Third-harmonic generation microscopy (45)	1 cross-sectional	Nonlinear optical response of biological tissues, with melanin providing strong THG contrast in human epidermis	High-resolution; real-time imaging; non-toxic; cost-effective compared to another microscopy	Lack of standardization; limited availability; lack of specificity; small sample size; limited information on deep structures
	Ultraviolet light photography (46–52)	4 cross-sectional; 3 case series	Ultraviolet light emitted at wavelengths of 320–450 nm (peak 365 nm) to improve visual contrast. Melanin in the epidermis absorbs UV rays more selectively than visible light.	Improved accuracy compared to visible light; cost-effective; painless; easy to use; real-time results; portable; non-radiative	False negative results, particularly in darker skin tones; operator dependence; may be limited by lighting conditions; uneven skin color may make it difficult to assess the extent and distribution of depigmented skin
	Visible light and digital photography (53, 54)	2 cross-sectional	Capture and storage of digital photograph	Visual database; potential use in telemedicine	Limited color accuracy; interobserver variability; image quality affected by lighting conditions, technical ability; need for further validation
Biophysical	Dermoscopy (46, 49, 55–63)	8 cross-sectional; 2 case series; 1 cohort	Transillumination of a lesion to investigate with high magnification (usually 10-fold) to visualize subtle colors, features, and microstructures in epidermis and papillary dermis	Cost-effective; real-time; reduced surface shining; easily applicable; improved diagnostic accuracy; early detection; increased patient satisfaction; Improved inter-observer agreement; better patient education; avoidance of pressure artifacts (no fluid immersions needed)	Operator dependence; limited specificity; limited information on deep structures; difficulty with dark skin; training needed for image interpretation; no quantitative analysis; interobserver variability;
	Colorimetry (64, 65)	1 cross-sectional; 1 case series	Quantifies skin color and evaluates pigmentation response to treatment, based on the principle of Beer-Lambert's Law	Standardization; quantification of color; suitable for all skin types; portable; safe	Limited sensitivity and specificity; potential observer bias; interference from adjacent pigmented areas; regular calibration needed
	Spectrometry (66–71)	5 cross-sectional; 1 cohort	Assess disease activity in vitiligo with optical signals with high spectral resolution	Safe; portable; cost effective; objective measure; easy to use; quantifiable; ultrafast (< 10 seconds analysis time)	Limited sensitivity and specificity; potential observer bias; sensitivity to environmental factors; complexity

### 3.2. Imaging techniques

#### 3.2.1. Ultraviolet light photography

Ultraviolet (UV) light serves as a standard and extensively employed diagnostic tool for evaluating vitiligo. Among the reviewed studies, UV light was utilized as a diagnostic technique in seven studies, with Wood's lamp being the preferred choice in six of them (46–51, 55). The theory behind UV light photography suggests that UV rays are selectively absorbed by melanin in the epidermis compared to visible light. This theory is primarily discussed in the context of using UV cameras. However, when it comes to diagnosing vitiligo specifically, most studies relied on standard cameras that capture images within the visible spectrum only. Therefore, the prominent visualization of vitiligo in photography mainly results from enhanced skin fluorescence due to the absence of superimposed pigment, rather than direct UV absorption. The most frequently used device to diagnose vitiligo using UV light is Wood's light, also called a Wood's lamp. It is a handheld device that emits long-wave UV light (wavelengths of 320–450 nm, peak 365 nm) and is equipped with a magnifying lens to enable close examination of the skin (47). Other devices for UV assessment have also been reported, including softboxes, camera flash, and high output flash. Uitentuis et al. discovered that varied UV set-ups produced significantly different quality images, with the high output flash technique producing the best characteristics for vitiligo assessment (47).

Furthermore, one study conducted by Kaliyadan et al. utilized a simple hand-held black-light source, specifically a rudimentary flashlight. This study revealed that the hand-held source was equally effective in detecting fluorescence or enhancing skin lesions, when compared to a standard Wood's lamp (55). Anbar et al. compared the accuracy of vitiligo lesion identification by dermatologists and patients with and without Wood's lamp (50). They observed that lesions outlined using Wood's lamp were significantly more prominent, leading to the detection of new clinically unseen lesions or the identification of expansions of clinically apparent lesions that were otherwise invisible under regular lighting conditions (50). Furthermore, the effectiveness of UV photography in monitoring disease stability has been demonstrated in multiple studies. For instance, Wang et al. conducted a study where Wood's lamp was utilized to assess vitiligo activity and disease stability, both crucial components for successful epidermal grafting surgery. The researchers observed that by examining amelanotic lesions with well-defined borders under Wood's lamp, which serves as an indicator of stability, dermatologists can identify suitable candidates for grafting. Moreover, the use of Wood's lamp facilitates the early detection of re-pigmentation, enabling accurate surveillance and evaluation of treatment outcomes (48).

#### 3.2.2. Reflectance confocal microscopy

Reflectance confocal microscopy (RCM) was used in eleven studies for vitiligo diagnosis and treatment monitoring (9–19). RCM performed *in vivo* is a non-invasive and repetitive imaging technique that generates real-time images with a resolution like histological images. The components of a reflectance confocal microscope include objective and condenser lenses, a detector, and a light source in the form of a near-infrared laser beam. The laser beam is directed to a particular spot on the skin. The microscope produces images from the stratum corneum to the upper dermis, with a maximum imaging depth of 250 μm and may be used as a non-invasive optical biopsy. RCM analysis facilitates recognizing typical characteristics of affected vitiligo skin and may be beneficial in distinguishing vitiligo in contrast to other hypopigmentary conditions such as post-inflammatory hypopigmentation, nevus depigmentosus, or nevus anemicus. The main RCM characteristics of vitiligo lesions observed throughout the studies included an apparent loss of melanin in the lesioned skin, an indistinct boundary separating the affected from the surrounding normal skin, loss of integrity of the bright dermal papillary rings generally seen at the dermo-epidermal junction level, and highly refractile inflammatory cell infiltration at the edge of the lesions. RCM has been shown useful to differentiate between active or stable vitiligo, proving to be useful in predicting stability in vitiligo. Pertaining to stable vitiligo, there is complete loss of melanin in affected skin, with no changes in the content of melanin nor the dermal papillary rings. In active vitiligo, there is also a loss of melanin alongside a disappearance of the dermal papillary rings (9).

#### 3.2.3. Computer-assisted imaging analysis

Currently, multiple approaches have been investigated for computer-assisted imaging analysis of vitiligo. The literature search found twenty studies which used variations of computer-assisted imaging analysis for vitiligo diagnostics.

Convolutional Neural Networks (CNNs) have shown promising results in vitiligo diagnosis and were utilized in eight studies (20–25). CNNs analyze skin images and classify them as either vitiligo or non-vitiligo lesions using a model involving multiple convolutional layers that identify patterns and a fully connected layer that performs the classification. A large dataset of labeled images is utilized to optimize the network performance and to minimize the difference between its predictions and the true labels.

Compared to human raters, including practicing dermatologists, dermatology residents, and general practitioners, CNNs outperformed all groups consistently. This suggests the efficacy of machine learning in classifying vitiligo by case probability and the possible advantages of CNN techniques as a remote diagnosis tool for vitiligo in situations involving telemedicine or where a Wood's lamp is not accessible (21, 26).

Principal component analysis (PCA) and independent component analysis (ICA) were proposed as mechanisms for analyzing vitiligo lesion segmentation and progression in five different studies (20, 27–30). While CNN, PCA, and ICA all use machine learning for image analysis, CNNs are non-linear supervised learning techniques, while PCA and ICA are linear unsupervised learning techniques. Generally, PCA is used to segment RGB images into melanin and hemoglobin only images, followed by ICA-powered alignment of the two principal component axes. In a study by Nugroho et al., a set of 41 RGB images of vitiligo lesions from 18 patients used a combinational PCA and ICA. They concluded that at a 95% confidence interval, there was a high sensitivity (0.9105 ± 0.0161), specificity (0.9973 ± 0.0009), and accuracy values (0.9901 ± 0.0028) (29). This diagnostic accuracy was comparable to other studies using PCA and ICA (20, 27–30, 72).

Two studies used Fuzzy C-Means (FCM), a cluster-based algorithm, to segment vitiligo lesions (31, 32). The algorithm follows a two-step mechanism, whereby skin segmentation is followed by vitiligo-specific segmentation. Both studies demonstrated considerably fast processing, illustrating the algorithm's promise for use in clinical settings and applicability for teledermatology applications. As the algorithm accepts low-resolution images as input, picture acquisition can be readily carried out using smartphone cameras. Furthermore, Nugraha et al. implemented the FCM software to develop a mobile application called Vi-DA (Vitiligo Diagnostic Assistance), allowing vitiligo patients to self-assess at home (32).

One study used artificial neural networks (ANN) of multilayer perceptron (MLP) type to quantifiable measure skin depigmentation based on the pattern of light refraction (33). Based on the light refraction pattern, the MLP was taught to analyze each pixel in an image and classify it as either having healthy or affected skin. The MLP then produces a binary picture from the original image, where cells with 0 and 1 represent healthy and vitiligo-affected skin, respectively. Compared to ICA/PCA and FCM, the proposed method outperformed both methods in specificity and sensitivity over an 8-test span (33).

Several studies demonstrated that computer-assisted imaging analysis provided quantitative data on vitiligo lesion stability, namely, consistent coloration over time, with little variation in pigmentation intensity, relatively constant lesions sizes, showing minimal or no expansion or contraction, and well-defined, regular borders, suggesting a lack of active disease progression.

#### 3.2.4. Optical coherence tomography

Four studies utilized Optical Coherence Tomography (OCT) for vitiligo assessment (38–41). OCT measures differences in optical path light, where one light path is directed to a tissue sample while the other is to a reference mirror (73). Through lateral scanning, OCT effectively constructs high-resolution two or three-dimensional cross-sectional images of microstructural morphology in biological tissue *in situ* (74, 75). A study by Su et al. demonstrated that OCT could effectively diagnose vitiligo in its early stages (40). This was accomplished by utilizing OCT for *in vivo* imaging to reconstruct a three-dimensional skin microstructure. This successfully identified any loss of pigment in the early stages of vitiligo, despite white patches of skin becoming prominent only in the later stages of the disease (40). Xie et al. came to a similar conclusion, supporting the efficacy of OCT in the early diagnosis of vitiligo (39).

Furthermore, detecting the stratum basale and dermal papillae is vital to diagnosing skin pigmentation disorders. To effectively identify such epidermal structures, OCT analyzes the low scattering property of the dermal papillae, contrasting it with the high scattering property of the pigmented basal layer (39). OCT's ability to autodetect such papillae structures are accomplished by scanning large areas of skin and assessing the decrease or absence of the scattering contrasting between the epidermal structures. Gao et al. studied OCT's ability to accurately quantify the optical path length, measuring *in vivo* tissue's refractive index (41). Their research demonstrated the ability of OCT to evaluate a lower refractive index in skin tissue with vitiligo, showcasing the contrast in scattering coefficients of skin with vitiligo versus without. This change in scattering coefficients was associated with a decrease in melanin content, thus confirming OCT's effectiveness in diagnosing vitiligo. Furthermore, OCT has shown promise in evaluating vitiligo stability. By analyzing the epidermal microstructure, OCT can identify well-defined lesion borders, preserved structural integrity, and relatively constant scattering coefficients in stable vitiligo cases. In contrast, unstable vitiligo was shown to exhibit irregular borders, disrupted epidermal architecture, and changes in scattering coefficients (41).

#### 3.2.5. Visible light and digital photography

Two studies investigated the use of visible light and digital photography for assessing vitiligo (53, 54). As vitiligo is primarily a clinical diagnosis, visible light and digital photography are essential tools for clinicians to use for educational, clinical or telemedicine applications. To better aid physicians in their assessments, digital photography's capture and storage help create a visual database of clinical presentations. The construction of robust visual databases is the foundation for advanced computational analysis to improve early diagnosis, monitor high-risk patients, and diagnose atypical lesions (53). For telemedicine, when imagining quality is consistent and adequate, it can substitute for inpatient physical examinations in up to 83% of cases (53). A recent article from Geel et al. highlights the importance of standardization in photographing vitiligo to improve documentation and comparison among different sites to produce a more efficient and reliable interpretation of results (54). Under visible light, distinguishing between hypomelanosis and amelanosis in vitiligo patients, particularly those with very fair skin (Type I or II) or children, has been shown to be challenging (76).

#### 3.2.6. Other techniques

Three more imaging techniques employed in vitiligo diagnosis include infrared photography, multiphoton microscopy and third-harmonic generation microscopy. Table 1 provides a summary of the detailed characteristics.

### 3.3. Biophysical techniques

#### 3.3.1. Dermoscopy

Dermoscopy, also known as epiluminescence microscopy, is a real-time, dynamic, nondiagnostic tool that enables *in vivo* examination of skin lesions. Clinically, dermoscopy is widely used in vitiligo diagnostics due to its ability to enhance visualization and detect subtle changes in pigmentation and skin lesions that may be difficult to discern with the naked eye. The technique involves using a handheld instrument equipped with a polarized light source and magnifying lens to visualize the epidermal layer's microstructures and the skin's superficial dermis. Dermoscopy can provide valuable information about the structure and appearance of affected vitiligo skin lesions, allowing for improved diagnostic accuracy.

Dermoscopy was utilized in eleven different studies (46, 49, 55–63), of which two studies used ultraviolet light (46, 49), and one used high dynamic range conversion of images for diagnostic and monitoring purposes (55). Perifollicular changes and interfollicular pigmentation constituted the most common dermoscopic observations seen by Al-Refu et al. and Jha et al. (56, 57). Two studies showed that employing UV-dermoscopy can identify various distinctive microscopic characteristics of vitiligo not seen in polarized dermoscopy, namely, an enhanced perifollicular border and a more distinguishable depigmented junctional zone (46, 49). Kaliyadan et al. observed that prominent pigmentary features were perceived to show significant enrichments after HDR conversion (55). Furthermore, a novel study by Scarfi et al. proposed utilizing fluorescence-advanced videodermatoscopy which enables dynamic examination of superficial skin structures with a cellular resolution and holds potential for improved disease monitoring, prognosis, and treatment outcomes in vitiligo patients (58). Dermoscopy also offers valuable insights into assessing disease stability in vitiligo by identifying key features such as indistinct boundaries, perifollicular depigmentation, satellite lesions, and the micro-Koebner phenomenon. These characteristics are significantly associated with active vitiligo. Conversely, the presence of perifollicular repigmentation serves as a promising indicator of vitiligo stabilization. These insights can assist in evaluating the activity of vitiligo lesions and provide useful guidance for patient counseling regarding disease prognosis.

#### 3.3.2. Colorimetry

The colorimeter is a non-invasive instrument used to quantify skin color and has also been utilized to determine the capacity for pigmentation. Colorimetry allows for objective quantification of epidermal changes associated with vitiligo and offers a standardized approach to evaluating disease stability. By precisely measuring parameters such as luminance value and melanin index, colorimetry provides valuable insights into the progression and response to treatment in vitiligo. Two articles illustrate the potential role and efficacy of the colorimeter as a non-invasive diagnostic tool for diagnosing vitiligo and its severity (64, 65). Brazzelli et al. used a portable colorimeter to evaluate the progressive development of vitiligo patches in a Caucasian male. The colorimeter evaluated an area of 8 mm^2^ in diameter of skin over four months, and they found the luminance value significantly increased, indicating increased relative lightness and depigmentation.

Tawfik et al. utilized colorimetry to evaluate the treatment response of vitiligo to narrow-band UVB phototherapy over the course of six months. The results were significant for regimentation in 90% of patients, as shown by an increase in melanin index, indicating improved stability of vitiligo lesions. An important finding was that the colorimeter could assess regimentation a month before it was apparent clinically, implicating its potential use as a prognostic tool (27). Compared to other measurement assessment techniques, such as the point counting method, colorimetry was found to be less time intensive and more standardized across all anatomic regions (27).

#### 3.3.3. Spectrometry

Six studies investigated the use of spectrometry in vitiligo assessment (66–71). These non-invasive tools can be used to assess disease activity in vitiligo patients. Spectrometers are portable tools used to investigate the physiological and morphological properties of skin tissue by utilizing optical signals with a high spectral resolution (72). The spectrometry mechanism of action involves exposing the epidermal surface to white light produced by an incandescent source. Melanin, one of the skin's chromophores, absorbs the majority of incoming light but exhibits a monotonic rise in intensity as wavelengths get shorter (absorption is almost wholly attenuated for wavelengths longer than 700 nm) (68). Spectrometers then detect backscattered photons emerging from various layers of skin tissue, resulting in a tissue surface emission profile. De Bruyne et al. used spectrometry on the perilesional skin of 70 vitiligo patients in different anatomic regions and noted a classification model generating a correct prediction in 82.9% of the cases (66). Poojary et al. analyzed using a portable fluorescence spectrometer for diagnosing vitiligo in 260 patients and recorded a similar sensitivity and specificity of 74.6% and 73%, respectively (67). They also observed a critical point (cut-off) of 975.995 nm to differentiate vitiligo from other hypopigmented with an increased sensitivity of 93.1% and specificity of 86.4%. Hegyi et al. utilized diffuse reflectance spectroscopy with an experimental spectrophotometer to measure skin pigmentation in patients with vitiligo undergoing PUVA therapy. They saw an increase in pigmentation compared to pre-treatment as demonstrated by a statistically significant difference in pre- and post-treatment melanin quantification (70). Park et al. utilized a narrow-band reflectance spectrophotometer to measure melanin indexes (Mis), relative melanin indexes (RMIs), and erythema to differentiate between two similar hypopigmented disorders, vitiligo and nevus depigmentosus (ND) (71). The study found that the mean Mis and RMIs are statistically different between patients with vitiligo and ND, with mean RMI scores of 50% and 74% for vitiligo and ND patients, respectively. Spectrometry techniques also offer a non-invasive means to assess vitiligo disease activity. Unlike invasive blood markers, spectrometry offers immediate and real-time information about the specific conditions of the skin and its biochemical alterations. Stability evaluation relies on monitoring the absence of significant changes in biochemical parameters and presents with distinctive benefits, including rapid analysis (<10 s), portability, and compactness, making it suitable for use in a dermatologist's office during consultations (66).

### 3.4. Quality assessment

Supplementary material 2 displays a visual representation of the methodological risk of bias assessment measured using the QUADAS-2 tool. The quality assessment of the included studies revealed a generally satisfactory level, as most studies demonstrated a low or unclear risk of bias. Notably, Domain 1 (Risk of Bias: Patient Selection) exhibited the highest risk, while Domain 2 (Risk of Bias: Index Test) demonstrated the lowest level of bias (Supplementary material 3).

## 4. Discussion

There is a significant need for non-invasive objective skin measurement methods that can accurately diagnose and monitor the progression of vitiligo. This systematic review analyzed 64 studies on various non-invasive diagnostic modalities, including eight imaging techniques, and three biophysical methods. This is especially useful for clinical practice but may also contribute to further research advancements.

Imaging techniques offer valuable insights into the structural changes in the epidermis that occur with vitiligo, which may contribute to a deeper understanding of the underlying pathogenesis. Another advantage of these techniques is their ability to provide highly detailed images and provide more accurate and objective information about the distribution and extent of depigmentation, aiding in earlier-stage diagnoses, which can be particularly important in detecting the progression of vitiligo and monitoring the efficacy of treatments (68). Using computer-assisted imaging analysis, particularly machine learning, is a relatively new and emerging field. Machine learning algorithms can be trained to analyze images of the skin and provide highly accurate assessments of the extent and progression of the condition. Unlike traditional imaging techniques, which rely on human interpretation, machine learning algorithms can provide consistent and unbiased assessments of the skin (26). This can be especially useful for large-scale studies and for tracking skin changes over time. Machine learning algorithms can be trained to identify specific patterns and features in images of the skin, which can be used to distinguish between different types of pigmentary disorders, which can be particularly important for the accurate diagnosis of vitiligo, as it is often misdiagnosed (77). However, there are also some limitations to the use of imaging techniques for the diagnosis of vitiligo. For reliable and repeatable results, imaging equipment can be expensive to acquire and requires stringent procedures to be followed by experienced professionals. Resolution and penetration depth are also restricted for imaging techniques, including RCM and infrared photography (12). Due to the lack of a capillary form standard, it may be challenging to quantify vessel abnormalities using these imaging techniques (14). Some imaging techniques, such as RCM and OCT, are not portable and are more expensive than other diagnostic modalities, straining accessibility for clinicians (39). Similarly, computer-assisted imaging analyses have shown promise in vitiligo diagnosis; however, they are limited in their dependence on training data and ability to identify rare or unusual cases (24).

One key advantage of biophysical methods such as spectrometry and colorimetry over imaging techniques is the ability to measure color and reflectance properties of the skin quantitatively, providing healthcare professionals with a more precise understanding of the extent and severity of pigmentation loss (66). Furthermore, all these techniques are portable, rapid, and objective measurement techniques. While biophysical techniques offer several advantages in diagnosing and assessing vitiligo, they also have some limitations that should be considered. One limitation is the sensitivity and specificity of these techniques, particularly colorimetry, and spectroscopy, which can be affected by factors such as skin hydration, skin oiliness, and skin temperature (78). To ensure accurate and reliable results, it is essential to standardize the conditions under which these measurements are performed and to use validated protocols and devices.

It is also pertinent to mention the available scales for assessing the extent and severity of vitiligo, each with its own strengths and limitations. These techniques combine visual assessment and quantitative measurements to evaluate the size, location, and progression of depigmented patches on the skin. The scores generated by these assessments play a crucial role in tracking the condition's progression and evaluating the effectiveness of treatment interventions. One commonly used technique is the Vitiligo Area Scoring Index (VASI), which divides the affected skin into four body regions and assigns a score based on the percentage of affected skin. However, it may not provide a comprehensive evaluation in cases of irregularly shaped patches or those located in hard-to-see areas (4). Another widely used metric is the Vitiligo Extent Score (VES). The VES assesses vitiligo in 19 body regions using template photographs, however, does not account for the back of the scalp, the soles of the feet, or the palms of the hands. and has shown higher dependability and usability compared to VASI. A simplified version of the VES, known as the Self-Assessment Vitiligo Extent Score (SA-VES), was developed as a patient-reported outcome measure. The SA-VES demonstrated excellent reliability and correlation with physicians' assessments, offering a user-friendly and practical approach for assessing vitiligo extent in clinical practice and research. In most validation studies included in this review, the VES showed higher dependability and usability compared to the Vitiligo Area Scoring Index (4–6, 79). The Vitiligo Disease Activity Score (VIDA) evaluates disease activity on a six-point scale based on patient self-reported assessments (80). While initially promising, a recent study by Coias et al. found the VIDA to be an unreliable assessment of disease activity, as VIDA scores did not correlate with changes in VASI scores over time, indicating patients' inaccurate prediction of disease activity (81). The Vitiligo European Task Force (VETF) assessment combines the evaluation of vitiligo extent, disease stage (staging), and disease progression (spreading). To assess the extent of vitiligo, the rule of nines, which is already utilized in atopic dermatitis assessment, is employed (82). In addition to these metrics, the Potential Repigmentation Index (PRI) is used to evaluate the extent of pigment loss, while the Patient-administered Vitiligo Screening Tool (VISTO) tracks symptoms and signs associated with vitiligo (83, 84). Recently, a novel metric called the Vitiligo Extent Tensity Index (VETI) has been proposed. The VETI score combines elements of VASI, VETF, and parts of PRI to create a more comprehensive system that offers reproducible numerical ratings and reduced interobserver variability (85). The point counting is a simple, precise, and useful technique that is frequently used to estimate irregularly shaped skin surface areas and was adapted for vitiligo by Aydin et al. (86).

The results of this systematic study should be evaluated considering various limitations and potential biases. Several studies in this systematic review had limited sample sizes, which impacted the generalizability of the results, hindering follow-up data from assessing the long-term effects of the different diagnostic methods. For practical linguistic reasons, English was the only language permitted for published studies, which has the potential to introduce language bias. Another limitation is that many of the studies provided an inadequate description of measurement sites, and there was often a lack of standardization and validation in the methods used. Moreover, due to the complexity of vitiligo symptoms, most methods could only measure a limited number of parameters. Subsequently, the information obtained may not be conclusive with a single diagnostic modality. Despite the abovementioned limitations, several methods exhibit encouraging potential for improved diagnosis and assessment, particularly in clinical and research contexts. Outside the widely used methods such as dermoscopy and Wood's lamp, RCT may be used as a non-invasive optical biopsy, computer-assisted imaging techniques can provide an effective remote diagnosis and allow patients to perform self-assessment at home, and spectroscopy and colorimetry are portable, rapid, and objective measurement techniques that may be particularly beneficial as an adjuvant to other diagnostic tools.

## 5. Conclusions

In conclusion, this systematic review summarizes the non-invasive imaging, biophysical, and manual methods now available for vitiligo diagnosis, severity evaluation, and therapeutic monitoring. While several of these techniques show promise and offer valuable insights regarding the structure and characteristics of vitiligo skin that cannot be obtained from the naked eye alone, suitable and verified protocols are required for further use of these tools in clinical and research settings. The systematic review provides valuable information for healthcare providers by offering a comprehensive summary of the available diagnostic techniques for vitiligo. The review results provide insight into each method's strengths and limitations and can inform clinical practice by guiding future research.

## Data availability statement

The original contributions presented in the study are included in the article/Supplementary material, further inquiries can be directed to the corresponding author.

## Author contributions

PA and MA contributed to the study design, including search strategy preparation, article screening, and data extraction and interpretation. PA, MA, CF, AC, AK, SQ, and HM were involved in drafting, revising, preparing the manuscript, and agreed to be accountable for all parts of the work and authorized the final version for submission. All authors contributed to the article and approved the submitted version.

## Supplementary material

Supplementary material 1 Keywords and search strategy (Ti, title; Ab, abstract; Kw, keywords; TS, topic (encompasses title, abstract, author keywords, keywords plus).

Supplementary material 2 QUADAS-2 risk-of-bias quality assessment presented for 64 studies.

Supplementary material 3 Weighted bar plots of the distribution of risk-of-bias judgements within each bias domain for included studies.
