# Supplementary material for: Non-invasive skin measurement methods and diagnostics for vitiligo: a systematic review
Source: Front Med (Lausanne). 2023 Jul 27;10:1200963. doi: 10.3389/fmed.2023.1200963 (PMC10416110; doi:10.3389/fmed.2023.1200963)
Supplement: Supplementary material 1 — Keywords and search strategy (Ti, title; Ab, abstract; Kw, keywords; TS, topic (encompasses title, abstract, author keywords, keywords plus). [file Table_1.docx]

**Tables:**

**Table 1:** Keywords and search strategy.
**Abbreviations:** Ti, title. Ab, abstract. Kw, keywords. TS, topic (encompasses title, abstract, author keywords, keywords plus)

| **Database** | **Search terms** |
| --- | --- |
| Scopus | [( TITLE-ABS-KEY ( vitiligo ) OR TITLE-ABS-KEY ( pigmentary AND disorder )] AND [TITLE-ABS-KEY ( diagnosis ) OR TITLE-ABS-KEY ( diagnostic ) OR TITLE-ABS-KEY ( detection ) OR TITLE-ABS-KEY ( assessment ) OR TITLE-ABS-KEY ( techniques ) OR TITLE-ABS-KEY ( tools ) OR TITLE-ABS-KEY ( sensitivity ) OR TITLE-ABS-KEY ( specificity ) OR TITLE-ABS-KEY ( screening ) OR TITLE-ABS-KEY ( accuracy ) OR TITLE-ABS-KEY ( predictive AND value ) ] |
| Cochrane | [(vitiligo):ti,ab,kw OR (pigmentary disorders):ti,ab,kw] AND [(diagnosis):ti,ab,kw OR (assessment):ti,ab,kw OR (techniques):ti,ab,kw OR (screening):ti,ab,kw OR (diagnostics):ti,ab,kw OR (detection):ti,ab,kw OR (sensitivity):ti,ab,kw OR (specificity):ti,ab,kw OR (predictive value):ti,ab,kw OR (accuracy):ti,ab,kw] |
| Embase | [(vitiligo):ti,ab,kw OR (pigmentary disorders):ti,ab,kw] AND [(diagnosis):ti,ab,kw OR (assessment):ti,ab,kw OR (techniques):ti,ab,kw OR (screening):ti,ab,kw OR (diagnostics):ti,ab,kw OR (detection):ti,ab,kw OR (sensitivity):ti,ab,kw OR (specificity):ti,ab,kw OR (predictive value):ti,ab,kw OR (accuracy):ti,ab,kw] |
| Web of Science | (TS=(vitiligo) OR TS=(pigmentary disorders)) AND (TS=(diagnosis) OR TS=(diagnostics) OR TS=(detection) OR TS=(assessment) OR TS=(techniques) OR TS=(tools) OR TS=(sensitivity) OR TS=(specificity) OR TS=(screening) OR TS=(accuracy) OR TS=(predictive value)) |
