# Supplementary figures and images for: Non-invasive skin measurement methods and diagnostics for vitiligo: a systematic review
Source: Front Med (Lausanne). 2023 Jul 27;10:1200963. doi: 10.3389/fmed.2023.1200963 (PMC10416110; doi:10.3389/fmed.2023.1200963)

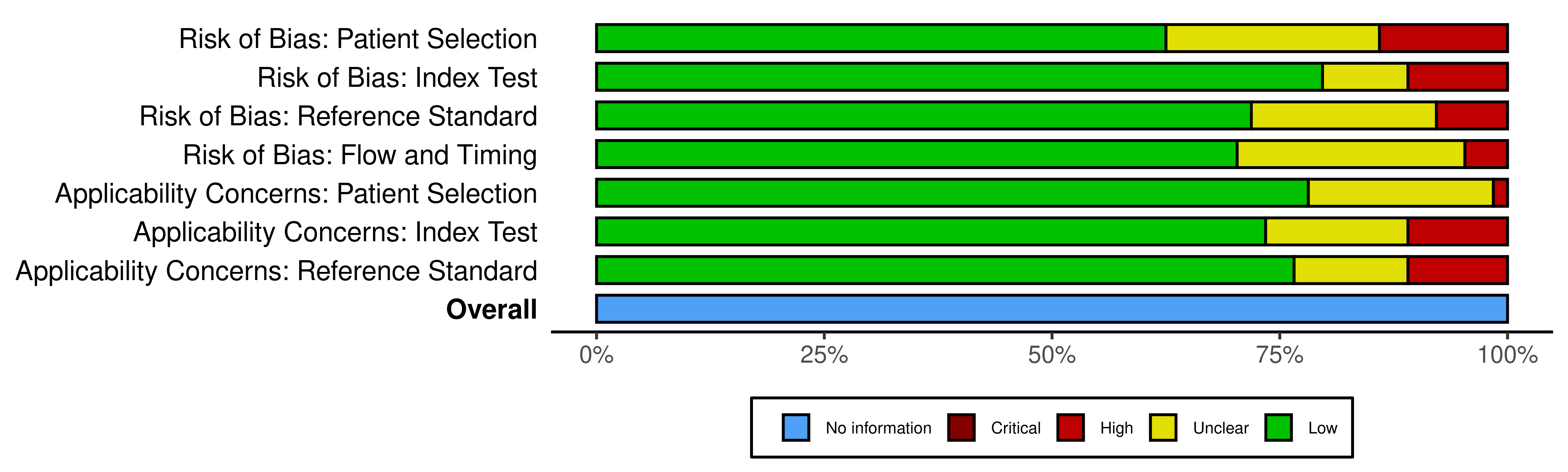

Supplement: Supplementary material 3 — Weighted bar plots of the distribution of risk-of-bias judgements within each bias domain for included studies. [file Image_2.png]
